# Fiber-optic hydrophone for detection of high-intensity ultrasound waves

**DOI:** 10.1364/OL.488862

**Published:** 2023-05-08

**Authors:** Esra Aytac Kipergil, Eleanor Martin, Sunish J. Mathews, Ioannis Papakonstantinou, Erwin J. Alles, Adrien E. Desjardins

**Affiliations:** 1 Department of Medical Physics and Biomedical Engineering, University College London, Malet Place Engineering Building, London WC1E 6BT, UK; 2 Wellcome/EPSRC Centre for Interventional and Surgical Sciences (WEISS), Charles Bell House, University College London, 43–45 Foley Street, London W1W 7TY, UK; 3 Photonic Innovations Lab, Department of Electronic and Electrical Engineering, University College London, Roberts Building, London WC1E 7JE, UK

## Abstract

Fiber-optic hydrophones (FOHs) are widely used to detect high-intensity focused ultrasound (HIFU) fields. The most common type consists of an uncoated single-mode fiber with a perpendicularly cleaved end face. The main disadvantage of these hydrophones is their low signal-to-noise ratio (SNR). To increase the SNR, signal averaging is performed, but the associated increased acquisition times hinder ultrasound field scans. In this study, with a view to increasing SNR while withstanding HIFU pressures, the bare FOH paradigm is extended to include a partially reflective coating on the fiber end face. Here, a numerical model based on the general transfer-matrix method was implemented. Based on the simulation results, a single-layer, 172 nm TiO_2_-coated FOH was fabricated. The frequency range of the hydrophone was verified from 1 to 30 MHz. The SNR of the acoustic measurement with the coated sensor was 21 dB higher than that of the uncoated one. The coated sensor successfully withstood a peak positive pressure of 35 MPa for 6000 pulses.

Fiber-optic hydrophones (FOHs) are widely used for the detection of high-intensity focused ultrasound (HIFU) fields for their robustness, large-bandwidth detection capabilities, and small footprints [1]. A typical FOH consists of a single-mode fiber with an uncoated end, which is cleaved perpendicularly to the fiber axis [2]. These FOHs rely on refractive index modulations of the fluid in front of the fiber tip, induced by pressure waves; the resulting changes in reflectance are monitored for ultrasound detection [2]. The manufacturing process of these FOHs is simple and cost-efficient, and they can be integrated with a basic optical detection setup [3]. However, a key challenge of using FOHs for ultrasound detection is their low signal-to-noise ratio (SNR) [4,5]. Although signal averaging can be performed to enhance SNR, the increased acquisition time limits ultrasound field scans. This challenge is significant when optical ultrasound transducers are the source: the lasers used for this purpose typically have low repetition rates (<50 Hz) [6]. In addition, the optical method of generating ultrasound results in high-frequency acoustic waves, which leads to a spatially confined focal spot (lateral width <100 μ m) [6]. Outside this region, higher signal averaging is required, for instance, during a search for the precise location of the focus. As a result of the low SNR, mapping ultrasound fields with an uncoated optical fiber can be very time-consuming.

The SNR of uncoated hydrophones can be improved by applying a partially reflective coating to the fiber end face [7–9], which increases the sensitivity to ultrasound. In this paradigm, homodyne [8] and heterodyne interferometers [9] have been used. However, these detection methods require more technical and signal processing effort, compared with the traditional reflection monitoring approach used with uncoated FOHs.

In this study, a coated FOH with a novel material, titanium dioxide (TiO_2_), is presented. The proposed hydrophone combines the advantages of both methods described: the high SNR of a coated hydrophone and a simple optical detection system. To model the uncoated and coated FOHs, a simulation framework based on the general transfer-matrix method [10] was implemented. These simulations provide insights into the underlying mechanisms of the coated hydrophones. Based on the simulation results, the hydrophones were fabricated, and their performance was evaluated experimentally.

The simulation framework based on the general transfer-matrix method explained by Katsidis and Siapkas [10] was developed and implemented in MATLAB (R2021a, Mathworks, USA). Using this model, the reflectance ( R ) as a function of pressure ( P ) was obtained. The derivative of reflectance with pressure ( dR/dP ), which is linearly proportional to FOH sensitivity [1,2] was calculated for the uncoated and coated hydrophones:

(1) $$\text{Sensitivity}=U_{dc}\left|\frac{dR}{dP}\right|\frac{1}{R+\alpha },$$

where $U_{dc}$ is the baseline DC voltage reading from the photodiode, and α accounts for nonidealities in the fiber-optic system. For the uncoated hydrophone, with the assumption of near normal incidence on the fiber end face and water interface [Fig. 1(a)] [1], the complex reflection coefficient ( $r_{unc}$ ) was calculated as [10,11]

(2) $$r_{unc}(P,\lambda )=\frac{n_{fc}(P,\lambda )-n_{w}(P,\lambda )}{n_{fc}(P,\lambda )+n_{w}(P,\lambda )},$$

where $n_{fc}$ and $n_{w}$ are the refractive indices of the fiber core and water, respectively, as a function of pressure ( P ) and optical interrogation wavelength ( λ ). For the coated hydrophone, the complex reflection coefficient ( $r_{c}$ ) was calculated as [10,11]

(3) $$r_{c}(P,\lambda )=\frac{r_{0,1}+r_{1,2}e^{-2i\delta_{1}}}{1+r_{0,1}r_{1,2}e^{-2i\delta_{1}}},$$

where $r_{0,1}$ and $r_{1,2}$ are the Fresnel reflection coefficients at the fiber end face–coating and coating–water interfaces [Fig. 1(b), Interfaces 0,1 and 1,2], respectively, and $\delta_{1}$ is the change in phase of the beam on traversing the coating, with the equations [10,11]

(4) $$r_{0,1}=\frac{n_{fc}(P,\lambda )-n_{c}(P,\lambda )}{n_{fc}(P,\lambda )+n_{c}(P,\lambda )},\;r_{1,2}=\frac{n_{c}(P,\lambda )-n_{w}(P,\lambda )}{n_{c}(P,\lambda )+n_{w}(P,\lambda )},$$

(5) $$\delta_{1}=\frac{2\pi n_{c}(P,\lambda )t(P)}{\lambda },$$

where $n_{c}$ and t are the refractive index and the thickness of the coating, respectively. The reflectance was obtained from the squared magnitude of the complex reflection coefficient ( $R=|r{|}^{2}$ ) [11].

**Fig. 1.**
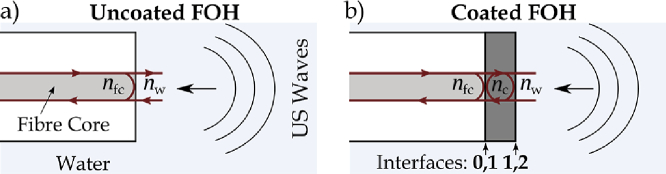
(a) Uncoated and (b) coated FOHs.

For the relation between the refraction index of water ( $n_{w}$ ) and pressure, the following equation, which combines the Tait equation and the Gladstone–Dale relation, was used [2]:

(6) $$n_{w}(P,\lambda )=1+(n_{w,0}(\lambda )-1){\left(1+\frac{P-P_{0}}{P_{0}+Q}\right)}^{1/\gamma }.$$

The Tait parameters are Q=295.5 MPa and γ=7.44 under static conditions ( $P_{0}=0.1$ MPa, $n_{w,0}=1.3177$ at λ=1565 nm) [2]. The changes in the refractive index and the thickness of the coating with a pressure variation were acquired from the following equations [8,12]:

(7) $$\Delta t=-\frac{\Delta P}{\rho \nu^{2}}t,\;\Delta n_{c}\approx \frac{0.3\Delta P}{\rho \nu^{2}},$$

where ρ and ν denote, respectively, the density and the speed of sound in the coating layer.

The relationship between the reflectance and the pressure can be assumed to be linear for both uncoated [13] and coated [12] hydrophones for pressure values between −30 and 100 MPa. Therefore, dR/dP is calculated from the derivative of the linear fit to R with respect to pressure.

For this study, ${\mathrm{TiO}}_{2}$ is considered as a coating material for its robustness, high mechanical stability, high adhesion on ${\mathrm{SiO}}_{2}$ , and high refractive index [14,15] (see Fig. S1 in the Supplement 1). Although higher sensitivity has been achieved with Fabry–Pérot polymer film hydrophones [16], the polymer spacer construction is not robust to high pressures and not suitable in this context [5]. The refractive indices of the fiber core ( $n_{fc}$ , fused ${\mathrm{SiO}}_{2}$ ,) and the coating ( $n_{c}$ , ${\mathrm{TiO}}_{2}$ ) were determined from their dispersion equations [17,18]. The simulation results were used to determine the specifications required for optimum sensor sensitivity at the optical wavelength of 1565 nm and guided the fabrication of single-layer ${\mathrm{TiO}}_{2}$ -coated FOHs.

Polyimide-coated single-mode fibers (SM1250(10.4/125)P, Fibercore, UK) were flat-cleaved using a cleaver (CT-30, Fujikura Ltd., Japan) for FOH fabrication. Fibers were positioned upward in a custom-made holder for coating. A single layer of ${\mathrm{TiO}}_{2}$ was coated on the fiber end face via plasma-assisted e-beam deposition (Helia Photonics, UK). The sensors have a flat surface, which is a natural outcome of cleaving a fiber at $90^{\circ }$ and simplifies the fabrication process.

Acoustic measurements were made to test the performance of TiO_2_-coated hydrophones in a tank filled with de-ionized and degassed water. Acoustic waves were generated by a single-element spherically focusing transducer (H101, Sonic Concepts, USA) with a diameter of 64 mm and a focal length of 62.5 mm. Input signals of three-cycle bursts at 3.3 MHz were produced by an arbitrary waveform generator (33611A, Keysight Technologies, USA) and amplified by a power amplifier (E & I A300 RF, Electronics & Innovation Ltd., USA) to drive the transducer. Generated signals were coupled to the transducer via an electrical impedance matching network and monitored using an oscilloscope (MSO-X 3104 T, Keysight Technologies, USA) via a 10X probe. To characterize the HIFU transducer [driven at the peak-to-peak voltage ( $V_{pp}$ ) of 46 V], an acoustic field scan was performed using a needle hydrophone (75 μ m; Precision Acoustics, UK) calibrated in the range 1–30 MHz. To detect ultrasound waves with an FOH, it was placed on a custom-made fiber holder and position-controlled using a three-axis motorized translation stage (MTS50/M-Z8, Thorlabs, Germany). The FOH was interrogated using a continuous wave laser (TSL-550, Santec Europe Ltd, UK) with a tuning range from 1500 to 1630 nm. The interrogation laser was operated at 1565 nm (the center of the tuning range) with an output power of 20 mW. The fluctuation of the power of the interrogation light source was ignored, as the voltage readings from a photodiode, which was added to the experimental setup, exhibited variations of less than 10% relative to the mean. An optical circulator was used to deliver the light to the fiber tip and return the reflected light to a photodiode (DET01CFC, 1.2 GHz, Thorlabs, Germany). The photodiode output was amplified by 10 dB via a preamplifier (DHPVA-200, Femto, Germany) and digitized via a data acquisition card (M4i.4420-x8, Spectrum, Germany) to monitor optical power modulation induced by pressure waves. The coated FOH was placed at the focus of the acoustic field, and acoustic waves were acquired with the transducer drive levels varying between 46 and 430 V. Pressure waves were high-pass filtered using an infinite impulse response Butterworth filter design with a frequency cutoff of 1 MHz. The same procedure was carried out using an uncoated hydrophone for comparison.

Figure 2 shows R and dR/dP calculated via the model for a single-layer ${\mathrm{TiO}}_{2}$ -coated hydrophone for coating thicknesses between 2 and 400 nm. Using the model, the coating thickness that results in the peak sensor sensitivity at the optical wavelength of 1565 nm was determined to be 166 nm. This value is close to the coating thickness of 172 nm, where the maximum reflectance occurs. For this reason, sensors were fabricated such that the coating would provide the maximum reflectivity, which can be easily monitored during FOH production.

**Fig. 2.**
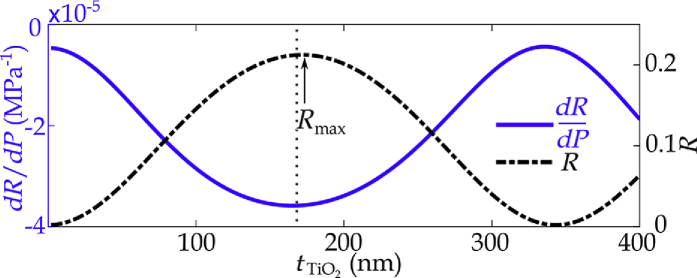
Plot of R and dR/dP for ${\mathrm{TiO}}_{2}$ -coated hydrophone. The dotted line corresponds to the coating thickness of peak sensitivity (166 nm). Peak reflectance ( $R_{\max}$ )= 172 nm.

The reflectance change with respect to the ambient pressure of 0.1 MPa for the pressure interval between −30 and 100 MPa was calculated for the uncoated and single-layer 172 nm ${\mathrm{TiO}}_{2}$ -coated FOHs at the optical interrogation wavelength of 1565 nm (see Fig. S2 in the Supplement 1); dR / dP of the coated hydrophone is calculated as 7.8× that of the uncoated one ( |dR/dP| : uncoated $\text{FOH}=4.62\times 10^{-6}$ , coated $\text{FOH}=3.59\times 10^{-5}$ ).

Three processes lead to the change in reflectance as a response to a variation of the acoustic pressure: changes in (i) the refractive index of water; (ii) the refractive index of the coating; and (iii) the thickness of the coating [12]. To understand the contribution of each component to the sensitivity of the sensor, the partial derivatives of reflectance with respect to pressure; keeping two of the variables of $n_{w}$ , $n_{c}$ , t constant (at ambient pressure); were calculated. These partial derivatives for the single-layer ${\mathrm{TiO}}_{2}$ -coated hydrophone were calculated for coating thicknesses ranging from 0 to 6 μ m. For a coating thickness up to around 4.3 μ m, the change in the refractive index of water [Fig. 3(a)], and for a coating thickness greater than 4.3 μ m, the thickness change of the coating [Fig. 3(c)] as a response to a variation in pressure becomes the major contributor to dR/dP ; in a thicker coating layer, the deformations [Eq. (7)] and the corresponding phase [Eq. (5)] and optical path length changes are larger for the same pressure variations. The sensitivity increases with the thickness of the coating layer; the greater change in the optical path length results in a higher dR/dP . However, realizing robust, micron-scale thicknesses could prove to be challenging in practice. Furthermore, the robustness of a thick coating and the frequency response of the hydrophones should be considered.

**Fig. 3.**
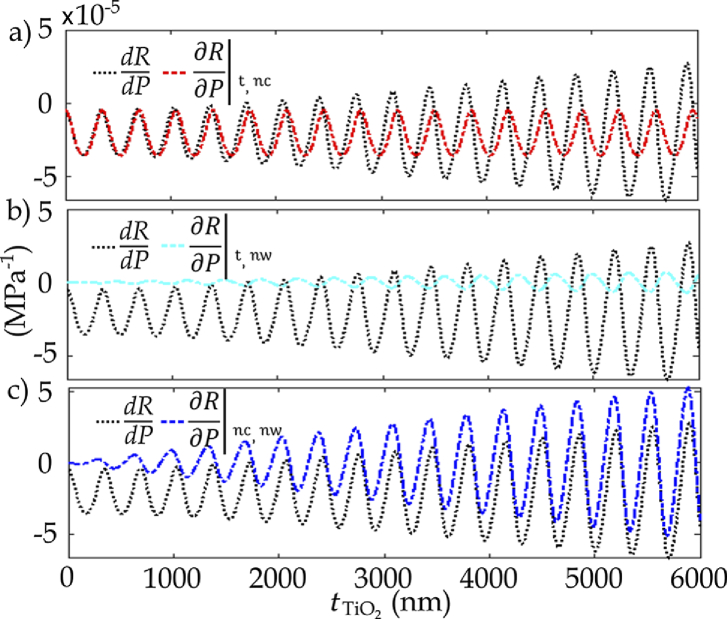
Plots of dR/dP and partial derivatives of reflectance with respect to pressure when: (a) $n_{c}$ and t ; (b) t and $n_{w}$ ; and (c) $n_{w}$ and $n_{c}$ are constant, as a function of ${\mathrm{TiO}}_{2}$ coating thickness.

Acoustic waveforms measured with a TiO_2_-coated hydrophone in the focal region of the transducer are shown in Fig. 4. The transducer was excited using three-cycle bursts at a peak-to-peak voltage of 120 V and a central frequency of 3.3 MHz. Signal averaging of 10 waveforms was performed. The measurements were taken at two different angles between the fiber tip and lateral–axial plane of the transducer, $0^{\circ }$ and $70^{\circ }$ , respectively [Fig. 4(a) and 4(b)]. Signal distortions were observed when the hydrophone was on-axis. The distortions are more visible in the second half of the waveform, where nonlinear effects are more strongly accumulated. Conversely, no such distortions were detected at the latter angle, potentially because of a more flat frequency response of the sensor, as reported by Krücker *et al.* [19]. Hence, the angled acquisition was adopted for acoustic measurements.

**Fig. 4.**
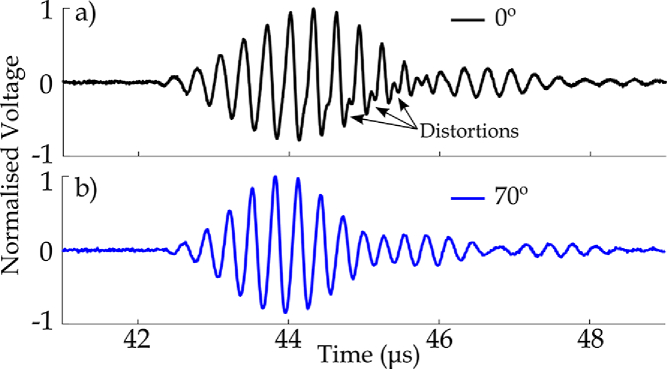
Measured acoustic waves at focus of HIFU field (10 averages, 3.3 MHz, three-cycle bursts, 120 V) with 172 nm TiO_2_-coated FOH; angle between fiber tip and lateral–axial plane of transducer: (a) $0^{\circ }$ ; (b) $70^{\circ }$ .

Two optical interrogation wavelengths, 1565 and 1610 nm, were used. The voltage amplitude of the acquired wave at 1610 nm was found to be 13% lower than that at 1565 nm. The refractive index of the coating is lower at 1610 nm than at 1565 nm [18], resulting in lower sensitivity of the FOH (see Fig. S3 in the Supplement 1).

Acoustic waveforms were acquired with the uncoated and TiO_2_-coated hydrophones at the focus of the field, which was driven at 46 V. The SNR of the measurement with the coated sensor was calculated to be 21 dB higher than that of the uncoated one (see Fig. S4 in the Supplement 1).

Pressure waves and spectra measured using the TiO_2_-coated hydrophone at the focus of the transducer driven at 46 and 378 V are shown in Fig. 5. The data show the acoustic waveform becoming increasingly nonlinear as the drive level increases, with increasing amplitude of the harmonics visible in the spectra.

**Fig. 5.**
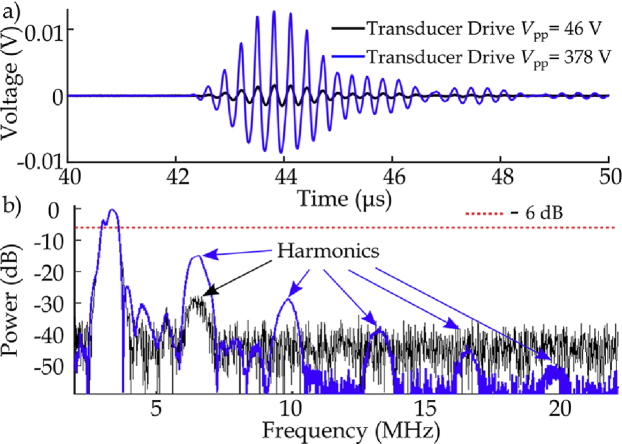
(a) Measured acoustic waveforms and (b) frequency spectra of acoustic measurements (10 averages, 3.3 MHz, three-cycle bursts, 46 and 378 V) acquired at the focus of the HIFU field with 172 nm TiO_2_-coated FOH.

The coated sensor endured a pressure of 35 MPa (peak positive) for 6000 pulses ( $V_{pp}=430$ V). Subsequently, measurements were repeated at lower pressures, and they matched the acquisitions taken prior to exposure to a peak positive pressure of 35 MPa. The magnitude of the pressure was obtained from the sensitivity of the hydrophone, which was determined from the measurements acquired at a low-pressure amplitude ( $V_{pp}=46$ V) by comparing the signal amplitudes with those obtained using a calibrated reference hydrophone, as described previously [6]. The sensitivity of the TiO_2_-coated hydrophones was found to be similar to those reported in previous studies on single-layer coated FOHs for high-intensity pressure wave measurement [7]. The frequency range of the hydrophone was verified from 1 to 30 MHz via an experiment conducted using a large-bandwidth optical ultrasound transducer and the calibrated reference hydrophone [20]. This response was assumed to be flat over a range of 1 to 12 MHz [19] at $70^{\circ }$ incidence. The drive voltage was kept below 430 V, to avoid damage to the transmitting transducer.

In previous studies, finite-element simulations and experiments showed that this response is determined by several factors, including the superposition of longitudinal, edge diffraction, lateral waves at the hydrophone front face, and a resonant vibration mode of the fiber body [21,22]. The relative contribution of each factor depends on the hydrophone configuration (single or multi-layer), the overall thickness, and the strain-optic coefficients of the layer or layers [21]. For single-layer coated FOHs (<300 nm), it has been found experimentally that the edge wave diffraction dominates the frequency response up to 12 MHz [22]. For a more uniform frequency response, different sensor geometries, such as a tapered fiber tip or rounded edges, can be utilized to suppress edge waves [13,21,23]. For future steps, rounded fiber tips will be considered. For thick coatings ( ≥25μ m), thickness-mode resonances contribute to the frequency response [21]. Once the frequency response of the FOH is known in amplitude and phase with angle, it can be deconvolved to recover the pressure waveform [21].

This study provided the following key innovations. Firstly, a novel material, TiO_2_, was evaluated as a coating material for FOHs to detect HIFU. Secondly, an elasto-optic numerical model was implemented to design and fabricate FOHs. These simulations revealed findings about single-layer coated hydrophones that have not been previously reported (to the best of the authors’ knowledge) including the value of dR/dP for the single-layer coated hydrophone, the optimum thickness required for maximum sensor sensitivity, the locations of consecutive dR/dP troughs (sensitivity peaks) for a coating thickness up to 6 μ m, and a detailed analysis of the contributors to the hydrophone’s response. Additionally, this study presented, for the first time, pressure measurements acquired at different wavelengths and angles between a coated FOH and the acoustic field. A primary limitation of the model implemented here is that it does not account for interactions between the ultrasound waves and the FOH; and therefore does not predict the frequency-phase response of the sensor. In future studies, multi-layer coatings could significantly improve the SNR. One consideration is that the frequency response of the multi-layer coated FOHs can be more complex than those with a single-layer coating, with pronounced resonant vibration and thickness modes [21].

## Data Availability

Data underlying the results presented in this paper are not publicly available at this time but may be obtained from the authors upon reasonable request.

## Supplemental document

See Supplement 1 for supporting content.
