# Supplementary material for: Fiber-optic hydrophone for detection of high-intensity ultrasound waves
Source: Opt Lett. 2023 May 8;48(10):2615–8. doi: 10.1364/OL.488862 (PMC10575604; doi:10.1364/OL.488862)
Supplement: Supplementary file 1 [file ol-48-10-2615-s001.pdf]

Supplemental Document

Optics Letters

Fiber-optic hydrophone for detection of high-intensity ultrasound waves: supplement

ESRA AYTAC KIPERGIL,[1,2,*] ELEANOR MARTIN,[1,2] SUNISH J. MATHEWS,[1,2] IOANNIS PAPAKONSTANTINOU,[3] ERWIN J. ALLES,[1,2] AND ADRIEN E. DESJARDINS[1,2]

[1] *Department of Medical Physics and Biomedical Engineering, University College London, Malet Place Engineering Building, London WC1E 6BT, UK*
[2] *Wellcome/EPSRC Centre for Interventional and Surgical Sciences (WEISS), Charles Bell House, University College London, 43–45 Foley Street, London W1W 7TY, UK*
[3] *Photonic Innovations Lab, Department of Electronic and Electrical Engineering, University College London, Roberts Building, London WC1E 7JE, UK*
[*] *e.kipergil@ucl.ac.uk*

This supplement published with Optica Publishing Group on 8 May 2023 by The Authors under the terms of the Creative Commons Attribution 4.0 License in the format provided by the authors and unedited. Further distribution of this work must maintain attribution to the author(s) and the published article's title, journal citation, and DOI.

Supplement DOI: https://doi.org/10.6084/m9.figshare.22633783

Parent Article DOI: https://doi.org/10.1364/OL.488862

Fibre-Optic Hydrophone For Detection of High-Intensity Ultrasound Waves: supplemental document

The supplementary figures are shown in this document.

The dR/dP values were calculated via simulations for a range of refractive indices of the coating between 2 and 3.7, and plotted as shown in Fig. S1. The refractive index of TiO_2 (2.27) is high and close to the ideal refractive index at which the absolute maximum of dR/dP, and thus, allow for a high sensitivity to be achieved.

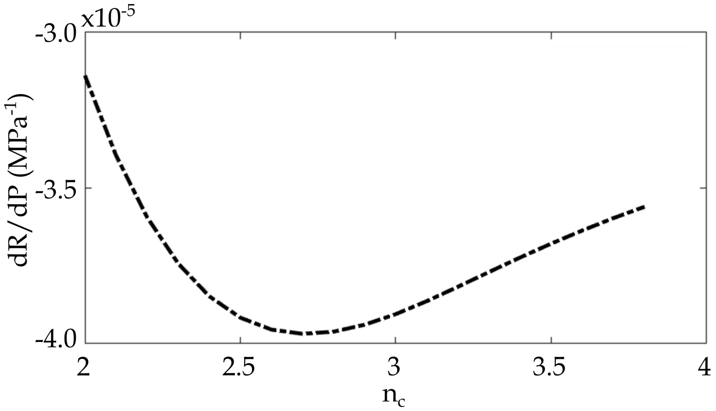

Fig. S1. dR/dP values acquired from simulations for a range of refractive indices of the coating. n_c: refractive index of coating.

The magnitude of the reflectance change at a given pressure is calculated to be higher for the coated hydrophone compared to an uncoated one (Fig. S2).

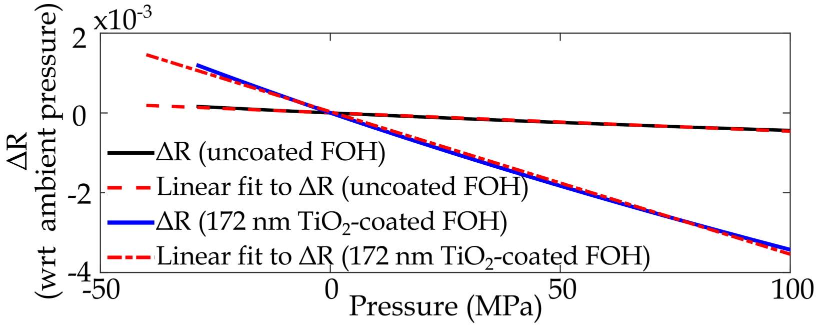

Fig. S2. Reflectance change (with respect to ambient pressure) versus pressure for the uncoated and TiO_2-coated FOHs.

Two optical interrogation wavelengths, 1565 and 1610 nm were used for acoustic measurements. The voltage amplitude of the acquired acoustic wave at 1610 nm was found to be 13% lower than the one at 1565 nm (Fig. S3). These two wavelengths (1565 and 1610 nm) were chosen for the following reasons: 1565 nm is the target optical wavelength for sensor fabrication as it is at the centre of the tuning range of the interrogation laser and 1610 nm is close to the end of the tuning range where the refractive index is lower (compared to 1565 nm) [1].

Fig. S4 shows the normalised acoustic waveforms acquired with the uncoated and TiO_2-coated hydrophones at the focus of the field, which was driven at 46 V. Signal averaging of one thousand waveforms was performed (due to the low SNR of the uncoated FOH) for both measurements

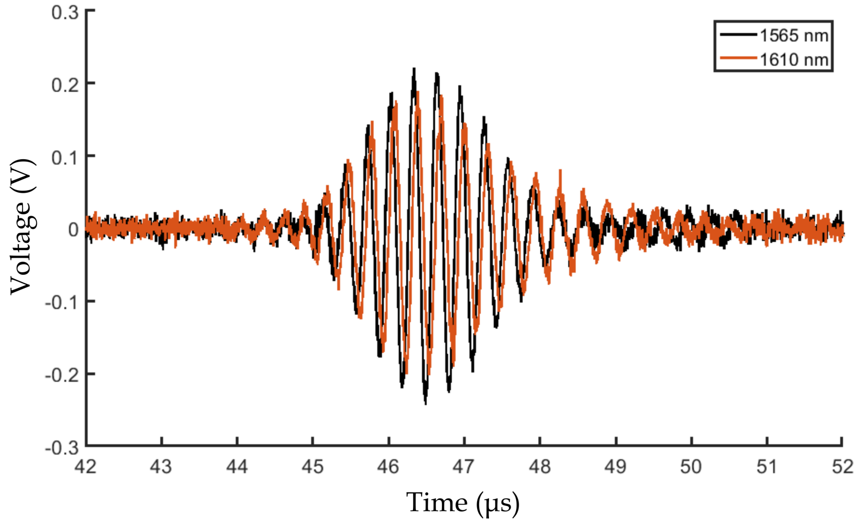

Fig. S3. Measured acoustic waves at the focus of a HIFU transducer (10 averages, 3.3 MHz, 3-cycle bursts, 46 V, 60 dB pre-amplification) with 172 nm TiO_2-coated FOH at the optical interrogation wavelengths of 1565 and 1610 nm.

and the waveforms were normalised for comparison purposes. The peak-positive pressure was measured as 3.1 MPa with the calibrated needle hydrophone at this drive voltage level. The SNR of the measurement with the coated sensor was calculated to be 21 dB higher than the case of the uncoated one.

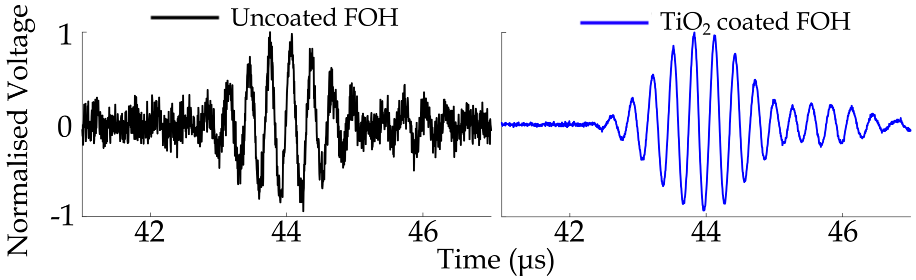

Fig. S4. Measured acoustic waves at the focus of the HIFU transducer (1000 averages, 3.3 MHz, 3-cycle bursts, 46 V) with the uncoated and 172 nm TiO_2-coated FOHs.

REFERENCES

1. J. R. DeVore, "Refractive indices of rutile and sphalerite," JOSA **41**, 416–419 (1951).
